# Eating occasion situational factors and sugar-sweetened beverage consumption in young adults

**DOI:** 10.1186/s12966-020-00975-y

**Published:** 2020-06-03

**Authors:** Sarah A. McNaughton, Felicity J. Pendergast, Anthony Worsley, Rebecca M. Leech

**Affiliations:** Institute for Physical Activity and Nutrition (IPAN), School of Exercise and Nutrition Sciences, Deakin University, Geelong, Australia

**Keywords:** Eating occasion, Eating patterns, Ecological momentary assessment, Situational factors, Sugar-sweetened beverage, Young adults

## Abstract

**Background:**

Young adulthood represents an influential transitional period marked by poor dietary habits and excess weight gain. Sugar-sweetened beverages (SSB) are a major source of excess caloric intake among young adults, yet little is known about the correlates of SSB consumption. This study examines the individual and situational correlates of SSB consumption, using real-time assessment of Australian young adults’ eating occasions.

**Methods:**

Dietary, sociodemographic and health behaviour data were collected during the Measuring EAting in Everyday Life (MEALS) study (*n* = 675 adults, 18–30 y). Participants reported all foods and beverages consumed over 3–4 non-consecutive days using a real-time Smartphone food diary application (“FoodNow”). For every eating occasion, food and beverage intake was recorded along with situational characteristics (eating location, purchase location, presence of others and activities while eating). A beverage occasion was defined as any eating occasion where a beverage was consumed and a SSB occasion was defined as any eating occasion where a SSB was consumed. Multilevel logistic regression was used to examine individual and situational characteristics with SSB intake at beverage occasions (i.e. factors associated with choosing a SSB over other non-alcoholic beverages) and to examine factors associated with consuming a SSB at any occasion where food and/or beverages were consumed.

**Results:**

Thirty-five percent of participants consumed SSBs during the recording period (*n* = 237). Of the 2185 beverage eating occasions reported by SSB consumers, 481 (20%) contained a SSB. SSB were rarely consumed on their own (i.e. other foods were present). Having a lower than tertiary education (odds ratio [95% confidence interval]: 1.53 [1.16, 2.01]; *p* < 0.01); eating in a café/restaurant, compared to at home (3.02 [1.58, 5.78]; *p* < 0.001), and purchasing beverages from a convenience outlet, compared to a supermarket/grocery store (4.58 [2.85, 7.38]; *p* < 0.001) were associated with SSB intake at beverage eating occasions. Similar associations were also found when all food and/or beverage eating occasions were examined.

**Conclusion:**

In this study, SSB were often consumed with other foods and intake was associated with individual and situational factors. Further studies are needed to confirm these findings and explore how SSB are consumed in relation to their accompanying foods.

## Background

There has been considerable attention on sugar-sweetened beverages (SSBs), the role they play in the diet and their implications for health outcomes. Systematic reviews of the evidence around the role of SSBs, and added sugars (of which SSBs are a major contributor) have shown that intake of free sugars or sugar-sweetened beverages is a determinant of body weight [1]. SSB intake has also been linked with diabetes, metabolic syndrome, some cardiovascular disease risk factors and dental caries [2–9]. Further, SSB intake is consistently associated with overall dietary patterns that are characterised by less healthy foods [10, 11].

Young adults are an important target group when considering the impact of poor nutrition on chronic disease risk, and for the development of nutrition promotion messages [12]. A recent modelling study of obesity trends in Australia demonstrates that young Australian adults are a major risk group for weight gain [13]. Young adults are now exposed to obesity and other risk factors earlier in life, resulting in a cumulative or longer lifetime exposure, and an increased risk of ill health [14, 15]. Young adulthood is characterised by multiple transitions such as changes in living, work and financial circumstances, and other major life changes (i.e. marriage and starting families) [16] which have the potential to impact on health behaviours such as food choice [17, 18]. Worldwide and compared to other adults, young adults are distinguished by poorer quality food consumption [19–21] are the highest consumers of SSBs [9, 22–24].

Determinants of eating behaviours have been extensively studied [25]. While there are many proposed models of the determinants of dietary behaviours, the more comprehensive approaches acknowledge that dietary behaviours are influenced by individuals’ interactions with their social and physical environments [25, 26]. Situational and contextual factors such as presence of others and eating away from home have been suggested to play a role in food intake [27–29] and evidence from a limited number of studies suggests that discretionary and energy-dense food and beverage choices may be especially prone to these influences [30–32]. Existing studies of correlates of SSB intake have predominantly focused on children and youth [33, 34] although studies in adults have identified screen time, as an important correlate [25]. Food purchasing environments have demonstrated mixed relationships with SSB consumption [35]. Foods and beverages are consumed multiple times per day in eating occasions [36], and maintainance of healthy dietary behaviours may vary due to changes in situational and contextual factors at individual eating occasions. Existing studies largely focus on overall measures of both dietary behaviours and influences, neglecting within- and between-day variation. Examining food intake at the level of the eating occasion, and the situational or context factors at each eating occasion, provides a novel framework for examining determinants of dietary behaviour.

Little is known about the situational correlates of SSB intake during individual eating occasions. A key reason for the paucity of research in this area is the widespread use of food frequency questionnaires where participants report their frequency of consumption of foods, but do not provide data on eating occasions. New technologies available via Smartphone “apps” and use of the principles of ecological momentary assessment techniques (EMA), [37, 38] allow real-time data collection in the settings in which the food is consumed [39] and allow the capture of data relating to situational factors [36, 39]. Understanding the drivers of SSB consumption in everyday eating situations will help inform the development of practical intervention strategies to modify behaviour and improve health outcomes. Therefore, the aim of this study was to examine individual and situational correlates of SSB intake during eating occasions in young adults.

## Methods

### Participants and procedures

This study used data obtained from the Measuring Eating in everyday Life Study (MEALS) which was designed to examine correlates of young adults eating patterns including patterning, format and context of eating occasions [36, 39]. For this cross-sectional study, participants aged 18–30 years and living in Victoria, Australia were recruited between April 2015 and April 2016 using social media (Facebook, twitter) and advertising at a range of sites and locations relevant to this age group (e.g. Universities, Institutes of Technical and Further Education [TAFE], shopping complexes). After providing written consent, participants were asked to complete an online questionnaire (Qualtrics online survey tool) and a Smartphone food diary (“FoodNow” Smartphone application) [38]. Participants were sent reminder emails and SMS messages to ensure completion of the online survey and food diary. They were compensated for their time with a shopping voucher ($25 per person). Ethical approval was granted by the Deakin University Human Ethics Advisory Group, Faculty of Health in February 2015 (HEAG-H 11_2015).

### Measures

#### Dietary assessment

Participants completed the FoodNow Smartphone food diary application (“app”) on three to four non-consecutive days including one weekend day over a period of 2 weeks. Inclusion of a weekend day, use of non-consecutive recording days and multiple days of recording were chosen based on recommendations from previous literature and practical considerations of minimization of respondent burden [40–42]. Participants were sent access to the Smartphone food diary and an instructional video link that contained information on how to download, log in and use the food diary. A full description of the FoodNow app is provided elsewhere [38].

Briefly, for each eating occasion, participants were asked to record the foods and beverages consumed by providing an image of the eating occasion and a short written description message of the types and amounts of each food or beverage consumed. For each eating occasion, participants were asked a number of questions relating to food acquisition and food preparation (e.g. who prepared and purchased the food items, cooking methods and meal preparation time) and situational factors (described in detail below). Reminders/prompts to use the FoodNow app were sent via ‘push’ notifications if participantsthey had not recorded any eating occasions within a three-hour period during waking hours (9 AM-9 PM). The day following a reporting day, participants were prompted to complete questions regarding dietary supplements, whether the previous day reflected usual consumption, skipped eating occasions or eating occasions which were consumed but not reported. The FoodNow Smartphone food diary was piloted [43] and then evaluated comparing energy intake against objectively measured energy expenditure with the target age group using SenseWear Armband (BodyMedia Inc., USA); it has shown good agreement between these methods [38].

Coding of the dietary intake data required matching each food and beverage item reported to an appropriate item in the 2011–2013 Australian Food and Nutrient Database (AUSNUT 2011–2013 [44], described in detail in [38]). The image and text description along with responses to questions about food preparation was used to match all foods consumed to a food or beverage item in the AUSNUT database, and to estimate the portion size. Coding of the data was completed by three trained nutritionists and data was checked for accuracy through a duplicate review process.

#### SSB intake

SSBs were defined according to standard Australian food composition database groups (5-digit food code level) and included regular (i.e. sugar-sweetened): flavoured soft drinks, flavoured mineral waters, commercial fruit juice drinks (100% fruit juice excluded), cordials, energy drinks, sports drinks and flavoured sweetened beverage bases (AUSNUT five-digit codes: 11307–11,309, 11,401, 11,403, 11,501, 11,503, 11,505, 11,601–11,603, 11,703, 11,802 and 11,804) [44]. An “eating occasion” was defined as any occasion when any food or beverage was ingested [45]. A beverage eating occasion was defined as any eating occasion where a non-alcoholic beverage was consumed and a SSB eating occasion was defined as any eating occasion where a SSB was consumed. Beverage-only eating occasions were defined as any eating occasion where only non-alcoholic beverages were consumed (i.e. no food was consumed). The median daily total frequency of SSBs (i.e. number of times SSBs were consumed), the median daily total energy intake (kJ) from SSB and the median daily total frequency of SSB serves (1 serve = 600 kJ) were calculated.

#### Situational factors at eating occasions

When using the FoodNow app, in addition to recording food and beverage intake for each eating occasion, participants were asked a number of questions relating to situational factors (See Additional File 1 for full details). For each eating occasion, participants were asked to indicate: 1) persons present while eating (categorised as: alone, with friends, with other people – i.e. family, partner, colleagues); 2) activity while eating (nothing else, visiting family/friends, screen-based activity, other activity); 3) location of eating (home - including family/friends’ home, work or university, fast food venue, café or restaurant, other location); and 4) location of food/beverage purchase (supermarket or grocery store, convenience type outlet, other location). These measures were based on previously developed questions by Laska et al. [46].

#### Sociodemographic and health behaviours

During an online questionnaire, participants were asked to report their age, gender and country of birth (categorised as Australia or other country). Highest level of formal education was measured using seven categories, ranging from ‘no formal qualification’ to ‘Higher University degree’, and, based on the distribution of responses, was categorised as a binary variable: less than tertiary education or tertiary education. Gross weekly household income was self-reported by participants and, based on the distribution, were categorised as: <$120, $120–499, $500–599 or ≥ $1000. Participants were asked to describe their main daily activity and/or employment status and their responses were categorised as: working full-time, working part-time, studying (full or part-time), or other (i.e. home duties, unemployed or looking for work). Participants’ living situations were reported and their responses were categorised as: with parents or family, alone, with partner or spouse, with a flatmate or friend, or other (i.e. with children, university/college residence). Participants reported their current relationship status and their responses were categorised as: married/in a defacto relationship (i.e. living together), dating/in a relationship, or not currently in a relationship. Smoking status was self-reported; based on the distribution of responses, participants were categorised as a never smoker or former/current smoker. Anthropometric measures (height and weight) were self-reported and body mass index (BMI) was calculated as weight (kg)/height (m^2^). Participants were then classified as healthy weight or overweight/obese (BMI ≥ 25 kg/m^2^).

#### Analytic sample

At the study completion, 842 eligible participants had completed the online survey, of whom 764 had completed at least one entry on one allocated recording day in the ‘Food Now’ App (Additional file 2). Eighty nine participants were also excluded because they had fewer than three non-consecutive days of dietary assessment, leaving a final analytic sample of 675 young adults (181 men and 499 women).

#### Data analysis

Group means (standard deviation) and medians (inter-quartile range) were used to summarise the sociodemographic, health behaviour and SSB consumption characteristics of participants. Independent t-tests (or the non-parametric equivalent, Wilcoxon Rank Sum test) and Pearson’s chi-square test were used to examine differences in sociodemographic and health behaviour characteristics between consumers and non-consumers of SSBs, for continuous and categorical variables, respectively. Differences in the characteristics of SSB consumption were examined overall and by gender using the Wilcoxon Rank Sum test. Frequency counts for the situational factors for all eating occasions where SSBs were consumed were calculated overall, and by gender, with gender differences tested using chi-square tests.

As the data structure had multiple eating occasions nested within individuals, multilevel regression analysis with random intercepts were used. Associations for eating occasion level (e.g. persons present, activity, eating location, purchase location [all categorical]) and individual level (e.g. age [continuous - grand mean centred], gender [binary], education level [binary], smoking status [binary], weight status [binary]) and factors with SSB intake at eating occasions were analysed. Two models were examined. The first model included only beverage eating occasions in order to examine factors associated with choosing a SSB over other non-alcoholic beverages. The second model included all eating occasions (i.e. both beverage eating occasions and food-only eating occasions) in order to examine factors associated with SSB intake at any occasion. All variables were entered simultaneously in each model and accounted for the correlated nature of the eating occasion level variables by correlating the residuals. Country of birth, income and marital status were also considered as covariates but their inclusion did not materially affect the results and so were removed. All data management and descriptive analyses were conducted using Stata statistical software, version 15.1 (Stata Inc., Texas, USA) and all multilevel regression analysis were conducted in M-Plus Version 7.31 (Muthen & Muthen, Los Angeles, CA, USA).

## Results

During the 3–4 day recording period, participants reported on average 4.6 (SD = 1.8) eating occasions per day. Overall, 35% of participants (*n* = 237) consumed SSBs during the recording period (Table 1), referred to herein as SSB consumers. There were few significant differences in the examined characteristics between consumers and non-consumers. Consumers of SSBs were more likely to have not have completed a tertiary degree and been born in Australia. They also had a significantly higher BMI, and a significantly greater proportion were categorised as overweight/obese, compared to non-consumers. When examining SSB consumption and energy intake contribution from SSBs among consumers (Table 2), there was no significant difference between the median frequency of consumption between men and woman, however men consumed a higher median number of serves and amount, in grams, of SSBs, compared to woman (both *P* = 0.001). Total energy intake and percentage of total energy intake from SSBs were also significantly higher for men than women (both *P* = 0.001). Sex differences were also observed according to the daily consumption frequency of eating occasions of other beverages (*P* < 0.001), but not SSBs (*P* = 0.32). SSBs were rarely consumed in isolation, as indicated by the low median frequency intake of beverage-only eating occasions containing SSBs.

**Table 1:** Characteristics of consumers and non-consumers of SSBs among Australian adults aged 18–30 years (*n* = 675)

n (%)	Consumers	Non-consumers	***P*** value^**a**^
	237 (35)	438 (65)	
Age, mean (95% CI)	24.0 (23.5, 24.4)	24.4 (24.1, 24.7)	0.14
Sex, n (%)			0.44
Male	59 (33)	121 (67)	
Female	178 (36)	317 (64)	
Country of birth			**0.015**
Australia	191 (38)	316 (62)	
Other	46 (27)	122 (73)	
Education level, n (%)			**0.042**
Lower than tertiary education^b^	110 (40)	168 (60)	
Tertiary education or higher	127 (32)	270 (68)	
Gross weekly household income, n (%)^b^			0.15
< $120	45 (34)	89 (66)	
$120–499	85 (37)	144 (63)	
$500–599	53 (41)	75 (59)	
≥ $1000	40 (29)	100 (71)	
Main daily activity, n(%)			0.19
Working full-time	67 (28)	137 (31)	
Working part-time	47 (20)	59 (13)	
Studying (full-time or part-time)	105 (44)	206 (47)	
Other	18 (8)	36 (8)	
Living situation, n (%)			0.58
With parents or family	82 (34)	143 (32)	
Alone	30 (13)	43 (10)	
With a partner or spouse	49 (21)	95 (22)	
With a flatmate or friend	67 (38)	145 (33)	
Other^c^	9 (4)	12 (3)	
Relationship status, n (%)			0.88
Married or in a defacto relationship	53 (37)	91 (63)	
In a committed relationship or dating	77 (35)	144 (65)	
Not currently in a relationship	107 (35)	203 (65)	
Smoking status			
Never smoker	193 (36)	350 (64)	0.63
Former/current smoker	44 (33)	88 (67)	
BMI score, mean (95% CI)^d^	23.3 (22.7, 23.9)	22.5 (22.2, 22.8)	**0.012**
Weight status, n (%)^e^			**0.027**
Healthy weight	169 (33)	348 (67)	
Overweight/obese	66 (42)	90 (58)	

^a^Differences between consumers and non-consumers assessed using a t-test for continuous variables and Pearson’s chi2 test for categorical variables

^b^Includes *n* = 86 (13%) participants who had completed a technical and further education diploma or certificate and/or a trade/apprenticeship and *n* = 178 (64%) of participants who were currently studying

^c^*n* = 631 with no missing data for income

^d^Includes responses “with children” (*n* = 17), university/college residence (*n* = 4)

^e^*n* = 673 with no missing data for BMI; results are geometric means (95% CI)

**Table 2:** Descriptive statistics of daily SSB consumption in consumers of SSBs, overall and by sex^a^

	Overall (***n*** = 237)	Men (***n*** = 59)	Women (***n*** = 178)	***P*** value^**b**^
**Daily consumption overall**				
Total SSBs consumed, grams/d	98 (124)	117 (150) 114 (292)	95 (119)	**0.001**
Number of times SSBs consumed, freq./d	0.3 (0.4)	0.3 (0.4) 0.5 (0.8)	0.5 (0.4)	0.31
Number of serves SSBs consumed, freq./d^c^	0.3 (0.3)	0.3 (0.3)	0.2 (0.3)	**0.001**
Total energy intake from SSBs, kJ/d	157 (199)	198 (191)	144 (189)	**0**.**001**
Percentage of total energy intake from SSBs, %	2 (3)	3 (3)	2 (3)	**0.008**
Total energy intake from all beverages, kJ/d	664 (715)	782 (900)	656 (653)	0.09
Percentage of beverage energy intake from SSBs, %	27 (35)	39 (43)	24 (34)	**0.01**
**Daily consumption by eating occasion**				
Eating occasions containing SSBs, freq./d	0.3 (0.3)	0.3 (0.3)	0.5 (0.3)	0.32
Eating occasions containing other beverages, freq./d	2.0 (1.5)	1.5 (1.5)	2.3 (1.5)	**< 0.001**
Beverage-only occasions, freq./d	0.8 (1.0)	0.5 (1.0)	0.8 (1.0)	0.05
Beverage-only occasions containing SSBs, freq./d	0 (0.3)	0 (0.3)	0 (0.3)	0.53

*Abbreviations*: *d* day, *EI* Energy intake, *freq*. frequency, *SSB* Sugar sweetened beverage

^a^Values are median (interquartile range)

^b^Wilcoxon rank sum test of differences between men and women

^c^One serve = 600 kJ

Of the 4613 eating occasions reported by SSB consumers, 2433 (53%) included a beverage and 481 (20%) of these occasions, included an SSB. Table 3 examines the frequency distribution of situational characteristics for eating occasions where SSBs were consumed, overall and by sex. The majority (> 50%) of young adult men and women reported being alone while consuming SSBs. However, compared to women, men reported having SSBs more often with friends than did women, who had a higher frequency of consuming SSBs with their family/partner (*P* = 0.03). The majority of SSBs were consumed at home, with work/university the next most common location, and screen-based activities were the most common activity reported while consuming SSBs. The majority of SSBs that were consumed had been purchased at the supermarket and this was significantly higher for women than men (67 v 47%), who reported purchasing approximately a third of their SSBs from convenience stores, compared to only 15% of women (*P* < 0.001).

**Table 3:** Frequency distribution of contextual characteristics for all eating occasions (*n* = 481) where sugar-sweetened beverages were consumed^a^

	Overall	Men	Women	***P*** value^**b**^
Persons present^c^				**0.03**
Alone	249 (53)	59 (51)	190 (53)	
With friends	61 (13)	23 (20)	38 (11)	
With other people (e.g. family/partner)	159 (34)	33 (29)	126 (36)	
Location of SSB intake^c^				0.16
Home (including family/friends’ home)	265 (56)	54 (48)	211 (59)	
Work/University	84 (18)	22 (19)	62 (17)	
Fast Food Venue	21 (5)	8 (7)	13 (4)	
Café/Restaurant	40 (8)	10 (9)	30 (8)	
Other^d^	59 (13)	19 (17)	40 (11)	
Activity^e^				0.31
Nothing else	99 (21)	23 (20)	76 (21)	
Visiting family/friends	70 (15)	13 (12)	57 (16)	
Screen-based activity	216 (46)	51 (45)	165 (46)	
Other^f^	83 (18)	26 (23)	57 (16)	
Location of purchase^g^				**< 0.001**
Supermarket/grocery store	282 (62)	52 (47)	230 (67)	
Convenience outlet^h^	86 (19)	36 (33)	50 (15)	
Other^i^	86 (19)	22 (20)	64 (19)	

^a^Values are n(%)

^b^Differences across gender assessed using Pearson’s chi2 test

^c^*n* = 12 occasions where persons present or location of sugar-sweetened beverage intake were not specified

^d^Includes sporting venue or in transit (i.e. car, bus train)

^e^*n* = 13 occasions where activity was not specified

^f^Includes in transit (i.e. car, bus train), reading/studying or playing sports

^g^*n* = 27 occasions where location of purchase was not specified/unknown

^h^Includes fast food/takeaway shop, Convenience store/milk bar/service station; vending machines

^i^Includes specialty shops, food markets, and common text responses stating ‘tap water’

Table 4 presents the individual and occasion level factors associated with SSB consumption at all beverage occasions, that is, those factors associated with choosing a SSB over other non-alcoholic beverages. Non-tertiary education, eating in a café/restaurant, rather than at home and purchasing beverages from a convenience outlet, rather than from a supermarket/grocery store, were associated with higher SSB intake (all adjusted odds ratios *P* < 0.01). In contrast, purchase location reported as “other”, compared to a supermarket/grocery store was associated with lower odds of SSB intake (*P* < 0.001). These associations were also observed when all eating occasions (i.e. beverage and food-only eating occasions) were included in the model **(**Table 5**).** However, eating with other people (e.g. family/partner), rather than eating alone, and eating at a fast food venue, rather than at home, were also negatively and positively associated with SSB intake, respectively, when all eating occasions were considered (both *P* < 0.05; Table 5).

**Table 4:** Individual and occasion level factors associated with consuming a SSB at eating occasions that include a beverage^a^

***Individual level: Sociodemographic and health behaviour factors***	
Age	1.00 (0.97, 1.02)
Gender	
Male (ref.)	1.00
Female	1.02 (0.72, 1.44)
Education level	
Tertiary or higher (ref.)	1.00
Less than tertiary	1.53 (1.16, 2.01)**
Smoking status	
Never smoker (ref.)	1.00
Past/current	1.26 (0.94, 1.68)
Weight Status	
Healthy/underweight (ref.)	1.00
Overweight/obese	1.20 (0.92, 1.57)
***Eating occasion level: Situational factors***	
Persons present	
Alone (ref.)	1.00
With friends	1.45 (0.94, 2.24)
With other people	0.83 (0.64, 1.07)
Location of SSB intake	
Home, including family/friends’ home (ref.)	1.00
Work/University	1.27 (0.89, 1.83)
Fast food venue	1.78 (0.77, 4.14)
café/restaurant	3.02 (1.58, 5.78)**
Other^b^	1.31 (0.86, 2.00)
Activity	
Nothing else (ref.)	1.00
Visiting family/friends	1.49 (0.89, 2.50)
Screen-based activity	1.21 (0.86, 1.71)
Other^c^	1.01 (0.70, 1.46)
Location of purchase	
Supermarket/grocery store (ref.)	1.00
Convenience outlet^d^	4.58 (2.85, 7.38)***
Other^e^	0.39 (0.26, 0.59)***

*Abbreviations*: *ref.* reference group, *SSB* Sugar-sweetened beverage

^a^ Values are odds ratios (95% CI) determined using multilevel logistic regression adjusted for all individual and eating occasion level factors (*n* = 2185 beverage EO with complete data on EO level situational factors)

^b^Includes sporting venue or in transit (i.e. car, bus train)

^c^Includes in transit (i.e. car, bus train), reading/studying or playing sports

^d^Includes fast food/takeaway shop, Convenience store/milk bar/service station; vending machines

^e^Includes specialty shops, food markets, and common text responses stating ‘tap water’

**P < 0.01, ***P < 0.001

**Table 5:** Individual and occasion level factors associated with consuming a SSB at all eating occasions^a^

***Individual level: Sociodemographic and health behaviour factors***	
Age	0.99 (0.98, 1.02)
Gender	
Male (ref.)	1.00
Female	1.01 (0.75, 1.36)
Education level	
Tertiary or higher (ref.)	1.00
Less than tertiary	1.29 (1.01, 1.64)*
Smoking status	
Never smoker (ref.)	1.00
Past/current	1.27 (0.98, 1.64)
Weight Status	
Healthy/underweight (ref.)	1.00
Overweight/obese	1.04 (0.83, 1.32)
***Eating occasion level: Situational factors***	
Persons present	
Alone (ref.)	1.00
With friends	1.10 (0.75, 1.60)
With other people	0.78 (0.62, 0.99)*
Location of SSB intake	
Home, including family/friends’ home (ref.)	1.00
Work/University	1.10 (0.80, 1.52)
Fast food venue	2.11 (1.08, 4.15)*
café/restaurant	2.85 (1.66, 4.89)***
Other^b^	1.35 (0.92, 1.98)
Activity	
Nothing else (ref.)	1.00
Visiting family/friends	1.09 (0.69, 1.71)
Screen-based activity	1.21 (0.89, 1.65)
Other^c^	1.23 (0.90, 1.75)
Location of purchase	
Supermarket/grocery store (ref.)	1.00
Convenience outlet^d^	3.32 (2.31, 4.78)***
Other^e^	0.58 (0.41, 0.81)**

*Abbreviations*: *ref.* reference group, *SSB* Sugar-sweetened beverage

^a^Values are odds ratios (95% CI) determined using multilevel logistic regression adjusted for all individual and eating occasion level factors (*n* = 4209 EO with complete data on EO level situational factors)

^b^Includes sporting venue or in transit (i.e. car, bus train)

^c^Includes in transit (i.e. car, bus train), reading/studying or playing sports

^d^Includes fast food/takeaway shop, Convenience store/milk bar/ service station; vending machines

^e^Includes specialty shops, food markets, and common text responses stating ‘tap water’

*P < 0.05, **P < 0.01, ***P < 0.001

## Discussion

This study examined SSB intake by eating occasions [36], to assess individual and situational factors associated with intake, such as eating location, purchase location, who else was present and what other activities were occurring. While some of these factors have been examined using 24-h recall and food diary methods in children [47], the examination of these factors simultaneously, as determinants of SSB intake during eating occasions in the current study is novel.

In the present study, 35% of young adults were SSB consumers, which is lower than the previous day consumption prevalence of 53% young adult men and 38% young adult women reported in the Australian Health Survey, 2011–13 [21]. We also observed that consumers of SSB were more likely to be born in Australia, had a higher BMI, and a higher proportion were categorised as overweight/obese, compared to non-consumers. Compared to women, men consumed more SSB in terms of daily energy intake and total amounts but not frequency. The frequency of beverage-only eating occasions containing SSBs was low and suggests that SSBs are consumed with other food items.

Comparisons with other studies are difficult due to the variety of dietary assessment methods used and different definitions of SSBs [23, 48]. For example, the Australian Health Survey and Victorian Health Monitor were conducted using 24-h recalls [21, 49], and a number of other relevant Australian studies have used frequency-based methods of assessment (food frequency questionnaires or short questions, with a variety of reference periods such as the previous year, previous month or previous day) [21, 49–52]. In addition, there is limited literature about young adults in Australia, with most studies in the last 10–15 years focusing on children and adolescents [47, 53].

Our findings showed that a large proportion of eating occasions containing SSBs occurred in the home which is consistent with findings relating to SSB consumption among Australian children and adolescents [47], and for other eating behaviours such as snacks [54]. Home food availability has been extensively studied as a determinant of food intake. However, the existing literature tends to focus on the impact on children and adolescents and families [55, 56] and other dietary behaviours such as fruit and vegetable intakes [57]. In one U.S study of young adults [58], living at home with parents or in rented accommodation was associated with poorer dietary habits and a lower availability of healthy foods, when compared to living on a university campus. In the present study, SSB consumption also did not differ by living situation and few participants reported living on campus, which is in line with national data that shows that less than 5% of Australian adults live on campus [59]. This suggests that situational factors determining food intake may not be generalizable across different countries and context-specific research is needed. Further, occupational and tertiary educational food environments are increasingly the focus of interventions in Australia, given the literature demonstrating the predominance of unhealthy foods and beverages present in tertiary education environments [60, 61].

In the present study, consumption of SSBs commonly occurred while undertaking screen-based activities (included watching TV/movies/cinema and using the computer) [36]. In contrast, a 7 day study of US women using ecological momentary assessment, Ghosh Roy et al. [62] found associations between snack consumption and watching television, but not SSB consumption however this sample was restricted to women and was not focused on young adults.

Associations between unhealthy food consumption and screen-based activities such as television viewing have been reported previously in the literature [60, 63–67]. However, many existing studies have relied on methods which did not capture the times when foods were consumed and so do not allow assessment of whether the two behaviours are occurring together, or the content of meals consumed while watching television. The current study provides evidence that these behaviours are occurring together rather than as a result of general clustering of unhealthy behaviours [68], however it is still possible there are common antecedents. While multiple mechanisms have been suggested for the associations between screen time and unhealthy food consumption including advertising and ‘mindless eating’ [69, 70], we are unable to identify cause and effect in this study, for example, whether selecting a particular activity drives consumption or vice-versa.

The majority of SSBs that were consumed had been purchased at a supermarket or grocery store, with women reporting more frequent SSB purchases from these locations than men. Convenience type outlets were also a popular purchase location among men. Our findings regarding purchase location are consistent with recently published results from the United States showing that supermarkets/grocery stores were the single largest source for SSBs [35, 71, 72], and previous results in Australian children and adolescents [47]. Whilst in the present study most SSBs were purchased from supermarkets, the odds of SSB consumption at eating occasions were higher when SSBs were purchased from a convenience store. This suggests that when young adults visited a convenience store they were more likely to select a SSB over other beverage choices at an eating occasion. Supermarkets are a major source of a wide variety of foods and beverages, which may explain why SSB consumption at eating occasions was not associated with purchasing from supermarkets.

In the present study, having a lower than tertiary education was the only individual factor associated with SSB intake at eating occasions. Direct comparisons with the literature are difficult to make as there are limited studies to draw on. However, these findings are consistent with the few existing studies of SSB consumption in young adults [20, 73, 74] and similar social gradients in dietary behaviours worldwide have been identified routinely [75, 76]. Consumption of SSBs has also been found to be associated with smoking in other studies [20, 73, 77]. However, in the present study, while current or past smokers had higher odds of SSB intake at eating occasions (OR = 1.29; *P* = 0.07), this association was not statistically significant although the proportion of current smokers in our study was low and were therefore grouped with former smokers.

When considering situational factors related to eating occasions, location of eating (café/restaurant, fast food venue) and purchase location (convenience outlet) were associated with higher odds of SSB intake at eating occasions. While direct comparisons are difficult, several studies have examined real-time situational factors associated with high energy snack food intake at eating occasions in adults [30, 31, 78]. The findings, however, are mixed. Proximity to fast food outlets but not to restaurants predicted high energy snacking in a sample of overweight or obese adults (*n* = 51) [30], whereas in other studies [78], proximity to fast food outlets or food shops was not associated with intake of high-energy snacks or SSBs [62].

A limitation of our study is the cross-sectional design which means we cannot determine cause and effect relationships between purchasing location and behaviour (that is, does location drive purchasing or does desire to consume drive choice of purchase location?). However, empirical studies have shown that SSB purchases can be influenced by store-level environmental factors such as product placement [79] and pricing [80], suggesting that these factors should be considered in further research and in public health interventions.

We found a modest association between the presence of other people (e.g. partner/family) and SSB intake and no associations were observed for activities while eating. While not directly comparable to our study, Schüz, Bower and Ferguson [31] found that the presence of family or partners was associated with lower odds of non-alcoholic beverage intake. In the same study, no associations were observed for leisure-time or work-related activities while eating. In contrast, observing others eat has been associated with intake of non-alcoholic beverages [31] and high-energy snacks [30, 78] which suggests that future research should not only measure who is present at eating occasions but also collect information on whether these persons were eating/drinking. A limitation of our study is that we did not include measures of other potential factors such as what and how much was consumed by other people present at the meal due to concerns about subject burden.

A key strength of this study was that it used real-time dietary assessment to examine SSB intake by eating occasions and the role of everyday situational factors, occurring during eating occasions, on SSB intake. Further, our sample was also large, relative to existing studies using EMA methods to assess dietary intake [46, 78, 81]. While dietary intake was self-reported, the Smartphone food diary was previously evaluated using objectively measured energy expenditure and there was good agreement between these methods [38]. While web-based recruitment has been shown to be an effective method of recruitment [82], recruitment of male participants proved difficult, resulting in a lower proportion of males in the final sample. The majority of participants had completed a tertiary degree or were currently studying and overall eating occasion frequency and SSB consumption were lower than levels reported in the Australian Health Survey. Therefore, our sample is unlikely to be representative of the broader young adult population and limits the generalisability of our findings. This may also impact on the interpretation of our findings in relation to correlates of SSB intake and further studies in more representative samples are needed to confirm these findings. Furthermore, a reduced sample completed 3–4 days of food diary resulting in a reduced sample size included in the final analysis, which may introduce bias. However, analysis of the original sample, the excluded participants and the final analytic sample suggests that there were no substantial differences with respect to important characteristics such as age and BMI [83]. Despite this, given the predominance of females, concerns about external validity are still relevant.

## Conclusions

This study of young adults showed that SSBs are predominantly consumed alone, at home, are purchased from supermarkets and consumed while undertaking screen-based activities. The study also found that SSBs are rarely consumed in isolation during eating occasions. Future research should explore the ways eating occasions containing SSBs differ from other beverage eating occasions, in relation to their accompanying foods to develop specific messages to promote healthier beverage choices. Finally, SSB intake was associated with individual and situational factors, which indicates the need to consider location specific messages and strategies to influence beverage choices. In summary, the findings suggest that interventions to modify SSB intake in young adults should target both the individual and their environment. However further studies are needed to confirm these findings.

## Supplementary information

**Additional file 1.** Questions (and corresponding response options) relating to situational factors during eating occasions that participants were asked while completing the ‘Food Now’ Smartphone Food Diary.

**Additional file 2.** Participant flowchart for the Measuring EAting in Everyday Life Study.

**Additional file 3.** A checklist of items included in the present manuscript as recommended by the extended STROBE-nut statement for nutritional observational studies [1, 2].

## Data Availability

The datasets used and/or analysed during the current study are available from the corresponding author on reasonable request.

## Abbreviations

AUSNUT: Australian Food and Nutrient Database
BMI: Body mass index
CI: Confidence interval
EMA: Ecological momentary assessment
MEALS: Measuring EAting in everyday Life Study
OR: Odds ratio
SSB: Sugar-sweetened beverage

## References

[1] Te Morenga L, Mallard S, Mann J (2012). Dietary sugars and body weight: systematic review and meta-analyses of randomised controlled trials and cohort studies. BMJ.

[2] Malik AH, Akram Y, Shetty S, Malik SS, Yanchou Njike V (2014). Impact of sugar-sweetened beverages on blood pressure. Am J Cardiol.

[3] Malik VS, Schulze MB, Hu FB (2006). Intake of sugar-sweetened beverages and weight gain: a systematic review. Am J Clin Nutr.

[4] Sonestedt E, Øverby NC, Laaksonen DE, Birgisdottir BE. Does high sugar consumption exacerbate cardiometabolic risk factors and increase the risk of type 2 diabetes and cardiovascular disease?. fnr [Internet]. 2012. [cited 2020 May 31];00. Available from:https://foodandnutritionresearch.net/index.php/fnr/article/view/496. 10.3402/fnr.v56i0.19104 PMC3409338 22855643

[5] Vartanian LR, Schwartz MB, Brownell KD (2007). Effects of soft drink consumption on nutrition and health: a systematic review and meta-analysis. Am J Public Health.

[6] World Health Organization (2015). Guideline: sugars intake for adults and children.

[7] Schulze MB, Manson JE, Ludwig DS, Colditz GA, Stampfer MJ, Willett WC, Hu FB (2004). Sugar-sweetened beverages, weight gain, and incidence of type 2 diabetes in young and middle-aged women. JAMA.

[8] O'Connor L, Imamura F, Lentjes MA, Khaw KT, Wareham NJ, Forouhi NG (2015). Prospective associations and population impact of sweet beverage intake and type 2 diabetes, and effects of substitutions with alternative beverages. Diabetologia.

[9] Imamura F, O'Connor L, Ye Z, Mursu J, Hayashino Y, Bhupathiraju SN, Forouhi NG (2015). Consumption of sugar sweetened beverages, artificially sweetened beverages, and fruit juice and incidence of type 2 diabetes: systematic review, meta-analysis, and estimation of population attributable fraction. BMJ.

[10] An R (2016). Beverage consumption in relation to discretionary food intake and diet quality among US adults, 2003 to 2012. J Acad Nutr Diet.

[11] Mullie P, Deliens T, Clarys P (2016). Relation between sugar-sweetened beverage consumption, nutrition, and lifestyle in a military population. Mil Med.

[12] Department of Health (2012). The Victorian health monitor.

[13] Hayes AJ, Lung TW, Bauman A, Howard K (2017). Modelling obesity trends in Australia: unravelling the past and predicting the future. Int J Obes.

[14] Abdullah A, Wolfe R, Stoelwinder JU, de Courten M, Stevenson C, Walls HL, Peeters A (2011). The number of years lived with obesity and the risk of all-cause and cause-specific mortality. Int J Epidemiol.

[15] Raitakari OT, Juonala M, Kahonen M, Taittonen L, Laitinen T, Maki-Torkko N, Jarvisalo MJ, Uhari M, Jokinen E, Ronnemaa T (2003). Cardiovascular risk factors in childhood and carotid artery intima-media thickness in adulthood: the cardiovascular risk in Young Finns study. JAMA.

[16] Anderson AS, Marshall DW, Lea EJ (2004). Shared lives-an opportunity for obesity prevention?. Appetite.

[17] Wethington E, Johnson-Askew WL (2009). Contributions of the life course perspective to research on food decision making. Ann Behav Med.

[18] Winpenny EM, van Sluijs EMF, White M, Klepp K-I, Wold B, Lien N (2018). Changes in diet through adolescence and early adulthood: longitudinal trajectories and association with key life transitions. Int J Behav Nutr Phys Act.

[19] McNaughton SA, Ball K, Crawford D, Mishra GD (2008). An index of diet and eating patterns is a valid measure of diet quality in an Australian population. J Nutr.

[20] Park S, Pan L, Sherry B, Blanck HM (2014). Consumption of sugar-sweetened beverages among US adults in 6 states: behavioral risk factor surveillance system, 2011. Prev Chronic Dis.

[21] Australian Bureau of Statistics (2014). Australian Health Survey: Nutrition First Results - Foods and Nutrients, 2011–12. Cat. No. 4364.0.55.007.

[22] Allman-Farinelli M, Partridge SR, Roy R (2016). Weight-related dietary behaviors in Young adults. Curr Obes Rep.

[23] Singh GM, Micha R, Khatibzadeh S, Shi P, Lim S, Andrews KG, Engell RE, Ezzati M, Mozaffarian D (2015). Global burden of diseases N, chronic diseases expert G: global, regional, and National Consumption of sugar-sweetened beverages, fruit juices, and Milk: a systematic assessment of beverage intake in 187 countries. PLoS One.

[24] Australian Bureau of Statistics (2018). Sugar sweetened drinks and diet drinks. National Health Survey: First Results, 2017–18. Cat. no. 4364.0.55.001.

[25] Sleddens EF, Kroeze W, Kohl LF, Bolten LM, Velema E, Kaspers P, Kremers SP, Brug J (2015). Correlates of dietary behavior in adults: an umbrella review. Nutr Rev.

[26] Glanz K, Bishop DB (2010). The role of behavioral science theory in development and implementation of public health interventions. Annu Rev Public Health.

[27] Bisogni CA, Falk LW, Madore E, Blake CE, Jastran M, Sobal J, Devine CM (2007). Dimensions of everyday eating and drinking episodes. Appetite.

[28] Robinson E, Blissett J, Higgs S (2013). Social influences on eating: implications for nutritional interventions. Nutr Res Rev.

[29] Larson N, Neumark-Sztainer D, Laska MN, Story M (2011). Young adults and eating away from home: associations with dietary intake patterns and weight status differ by choice of restaurant. J Am Diet Assoc.

[30] Elliston KG, Ferguson SG, Schuz N, Schuz B (2017). Situational cues and momentary food environment predict everyday eating behavior in adults with overweight and obesity. Health Psychol.

[31] Schuz B, Bower J, Ferguson SG (2015). Stimulus control and affect in dietary behaviours. An intensive longitudinal study. Appetite.

[32] Schuz B, Schuz N, Ferguson SG (2015). It's the power of food: individual differences in food cue responsiveness and snacking in everyday life. Int J Behav Nutr Phys Act.

[33] Sleddens EF, Kroeze W, Kohl LF, Bolten LM, Velema E, Kaspers PJ, Brug J, Kremers SP (2015). Determinants of dietary behavior among youth: an umbrella review. Int J Behav Nutr Phys Act.

[34] Barrett P, Imamura F, Brage S, Griffin SJ, Wareham NJ, Forouhi NG (2017). Sociodemographic, lifestyle and behavioural factors associated with consumption of sweetened beverages among adults in Cambridgeshire, UK: the fenland study. Public Health Nutr.

[35] Gustafson A, Christian JW, Lewis S, Moore K, Jilcott S (2013). Food venue choice, consumer food environment, but not food venue availability within daily travel patterns are associated with dietary intake among adults, Lexington Kentucky 2011. Nutr J.

[36] Leech RM, Worsley A, Timperio A, McNaughton SA (2015). Understanding meal patterns: definitions, methodology and impact on nutrient intake and diet quality. Nutr Res Rev.

[37] Shiffman S, Stone AA, Hufford MR (2008). Ecological momentary assessment. Annu Rev Clin Psychol.

[38] Pendergast FJ, Ridgers ND, Worsley A, McNaughton SA (2017). Evaluation of a smartphone food diary application using objectively measured energy expenditure. Int J Behav Nutr Phys Act.

[39] Pendergast FJ, Leech RM, McNaughton SA (2017). Novel online or Mobile methods to assess eating patterns. Curr Nutr Rep.

[40] Willett WC (1998). Nutritional Epidemiolgy.

[41] An R (2016). Weekend-weekday differences in diet among U.S. adults, 2003-2012. Ann Epidemiol.

[42] Hartman AM, Brown CC, Palmgren J, Pietinen P, Verkasalo M, Myer D, Virtamo J (1990). Variability in nutrient and food intakes among older middle-aged men. Implications for design of epidemiologic and validation studies using food recording. Am J Epidemiol.

[43] Pendergast F. Examining the eating patterns of young adults. Ph.D. Thesis: Deakin University; 2017. Accessed from: http://dro.deakin.edu.au/view/DU:30103489.

[44] Food Standards Australia New Zealand (2014). AUSNUT 2011–13 – Australian food composition database.

[45] Leech RM, Worsley A, Timperio A, McNaughton SA (2015). Characterizing eating patterns: a comparison of eating occasion definitions. Am J Clin Nutr.

[46] Laska MN, Graham D, Moe SG, Lytle L, Fulkerson J (2011). Situational characteristics of young adults’ eating occasions: a real-time data collection using personal digital assistants. Public Health Nutr.

[47] Hafekost K, Mitrou F, Lawrence D, Zubrick SR (2011). Sugar sweetened beverage consumption by Australian children: implications for public health strategy. BMC Public Health.

[48] Miller PE, McKinnon RA, Krebs-Smith SM, Subar AF, Chriqui J, Kahle L, Reedy J (2013). Sugar-sweetened beverage consumption in the U.S.: novel assessment methodology. Am J Prev Med.

[49] Department of Health & Human Services (2012). The Victorian health monitor food and nutrition report.

[50] French S, Rosenberg M, Wood L, Maitland C, Shilton T, Pratt IS, Buzzacott P (2013). Soft drink consumption patterns among Western Australians. J Nutr Educ Behav.

[51] Pollard CM, Meng X, Hendrie GA, Hendrie D, Sullivan D, Pratt IS, Kerr DA, Scott JA (2016). Obesity, socio-demographic and attitudinal factors associated with sugar-sweetened beverage consumption: Australian evidence. Aust N Z J Public Health.

[52] Shi Z, Taylor AW, Wittert G, Goldney R, Gill TK (2010). Soft drink consumption and mental health problems among adults in Australia. Public Health Nutr.

[53] Australian National Preventive Health Agency (ANPHA) (2014). Obesity: Sugar-Sweetened Beverages, Obesity and Health.

[54] Myhre JB, Loken EB, Wandel M, Andersen LF (2015). The contribution of snacks to dietary intake and their association with eating location among Norwegian adults - results from a cross-sectional dietary survey. BMC Public Health.

[55] Cook LT, O'Reilly GA, DeRosa CJ, Rohrbach LA, Spruijt-Metz D (2015). Association between home availability and vegetable consumption in youth: a review. Public Health Nutr.

[56] Mazarello Paes V, Hesketh K, O'Malley C, Moore H, Summerbell C, Griffin S, van Sluijs EM, Ong KK, Lakshman R (2015). Determinants of sugar-sweetened beverage consumption in young children: a systematic review. Obes Rev.

[57] Larson N, Laska MN, Story M, Neumark-Sztainer D (2012). Predictors of fruit and vegetable intake in young adulthood. J Acad Nutr Diet.

[58] Nelson Laska M, Larson NI, Neumark-Sztainer D, Story M (2010). Dietary patterns and home food availability during emerging adulthood: do they differ by living situation?. Public Health Nutr.

[59] Australian Bureau of Statistics (2013). Australian Social Trend, July 2013. Cat. No. 4102.0.

[60] Roy R, Hebden L, Kelly B, De Gois T, Ferrone EM, Samrout M, Vermont S, Allman-Farinelli M (2016). Description, measurement and evaluation of tertiary-education food environments. Br J Nutr.

[61] Roy R, Kelly B, Rangan A, Allman-Farinelli M (2015). Food Environment Interventions to Improve the Dietary Behavior of Young Adults in Tertiary Education Settings: A Systematic Literature Review. J Acad Nutr Diet.

[62] Ghosh Roy P, Jones KK, Martyn-Nemeth P, Zenk SN (2019). Contextual correlates of energy-dense snack food and sweetened beverage intake across the day in African American women: An application of ecological momentary assessment. Appetite.

[63] Laska MN, Hearst MO, Lust K, Lytle LA, Story M (2015). How we eat what we eat: identifying meal routines and practices most strongly associated with healthy and unhealthy dietary factors among young adults. Public Health Nutr.

[64] Leech RM, McNaughton SA, Timperio A (2014). The clustering of diet, physical activity and sedentary behavior in children and adolescents: a review. Int J Behav Nutr Phys Act.

[65] Leech RM, McNaughton SA, Timperio A (2014). Clustering of children's obesity-related behaviours: associations with sociodemographic indicators. Eur J Clin Nutr.

[66] Thorp AA, McNaughton SA, Owen N, Dunstan DW (2013). Independent and joint associations of TV viewing time and snack food consumption with the metabolic syndrome and its components; a cross-sectional study in Australian adults. Int J Behav Nutr Phys Act.

[67] Pearson N, Biddle SJ (2011). Sedentary behavior and dietary intake in children, adolescents, and adults. A systematic review. Am J Prev Med.

[68] Livingstone KM, McNaughton SA (2017). A Health Behavior Score is Associated with Hypertension and Obesity Among Australian Adults. Obesity (Silver Spring).

[69] Cleland VJ, Patterson K, Breslin M, Schmidt MD, Dwyer T, Venn AJ (2018). Longitudinal associations between TV viewing and BMI not explained by the ‘mindless eating’ or ‘physical activity displacement’ hypotheses among adults. BMC Public Health.

[70] Cox R, Skouteris H, Rutherford L, Fuller-Tyszkiewicz M, Dell’ Aquila D, Hardy LL (2012). Television viewing, television content, food intake, physical activity and body mass index: a cross-sectional study of preschool children aged 2-6 years. Health Promot J Austr.

[71] An R, Maurer G (2016). Consumption of sugar-sweetened beverages and discretionary foods among US adults by purchase location. Eur J Clin Nutr.

[72] Drewnowski A, Rehm CD (2013). Energy intakes of US children and adults by food purchase location and by specific food source. Nutr J.

[73] Larson N, Laska MN, Story M, Neumark-Sztainer D (2015). Sports and energy drink consumption are linked to health-risk behaviours among young adults. Public Health Nutr.

[74] Giskes K, Avendano M, Brug J, Kunst AE (2010). A systematic review of studies on socioeconomic inequalities in dietary intakes associated with weight gain and overweight/obesity conducted among European adults. Obes Rev.

[75] Olstad DL, Leech RM, Livingstone KM, Ball K, Thomas B, Potter J, Cleanthous X, Reynolds R, McNaughton SA (2018). Are dietary inequalities among Australian adults changing? A nationally representative analysis of dietary change according to socioeconomic position between 1995 and 2011-13. Int J Behav Nutr Phys Act.

[76] Drewnowski A (2009). Obesity, diets, and social inequalities. Nutr Rev.

[77] Zizza CA, Sebastian RS, Wilkinson Enns C, Isik Z, Goldman JD, Moshfegh AJ (2015). The contribution of beverages to intakes of energy and MyPlate components by current, former, and never smokers in the United States. J Acad Nutr Diet.

[78] Elliston KG, Ferguson SG, Schüz B (2017). Personal and situational predictors of everyday snacking: An application of temporal self-regulation theory. Brit J Health Psych.

[79] Huse O, Blake MR, Brooks R, Corben K, Peeters A (2016). The effect on drink sales of removal of unhealthy drinks from display in a self-service cafe. Public Health Nutr.

[80] Blake MR, Peeters A, Lancsar E, Boelsen-Robinson T, Corben K, Stevenson CE, Palermo C, Backholer K (2018). Retailer-led sugar-sweetened beverage Price increase reduces purchases in a hospital convenience store in Melbourne, Australia: a mixed methods evaluation. J Acad Nutr Diet.

[81] Zenk SN, Horoi I, McDonald A, Corte C, Riley B, Odoms-Young AM (2014). Ecological momentary assessment of environmental and personal factors and snack food intake in African American women. Appetite.

[82] Gosling SD, Vazire S, Srivastava S, John OP (2004). Should we trust web-based studies? A comparative analysis of six preconceptions about internet questionnaires. Am Psychol.

[83] Pendergast FJ, Livingstone KM, Worsley A, McNaughton SA (2019). Examining the correlates of meal skipping in Australian young adults. Nutr J.

