# Supplementary material for: Eating occasion situational factors and sugar-sweetened beverage consumption in young adults
Source: Int J Behav Nutr Phys Act. 2020 Jun 3;17:71. doi: 10.1186/s12966-020-00975-y (PMC7271392; doi:10.1186/s12966-020-00975-y)
Supplement: Supplementary file 1 — Additional file 1. Questions (and corresponding response options) relating to situational factors during eating occasions that participants were asked while completing the ‘Food Now’ Smartphone Food Diary. [file 12966_2020_975_MOESM1_ESM.docx]

**Additional File 1. Questions (and corresponding response options) relating to situational factors during eating occasions that participants were asked while completing the ‘Food Now’ Smartphone Food Diary**

| **Situational factor assessed** | **Question asked at every eating occasion** | **Response options** |
| --- | --- | --- |
| Person present while eating | Who were you with while eating/drinking? | By myself; with friends; with my partner; with my children; with other family members; with work colleagues; with other uni students; other (text response) |
| Eating location | What type of place were you at during this eating/drinking occasion? | Home; work; university; friend/family’s home; restaurant; coffee shop/café; fast food venue, sporting venue; in transit; other (text response) |
| Activity while eating | What were you doing while eating/drinking? | Nothing else, just eating; watching TV/movies/cinema; using the computer; in transit; talking/texting on phone; visiting or socialising with friends/family; reading/studying; playing sports; other (text response) |
| Purchase location | Where was this food or the main ingredients for the meal purchased? | Large supermarket; canteen; fast food/takeaway shop; restaurant/café/bar/club; local grocery store; specialty shop (butcher, bakery, green grocer); food market; Convenience store/ milk bar/ service station; vending machine; not sure; other (text response) |
