# Supplementary material for: Eating occasion situational factors and sugar-sweetened beverage consumption in young adults
Source: Int J Behav Nutr Phys Act. 2020 Jun 3;17:71. doi: 10.1186/s12966-020-00975-y (PMC7271392; doi:10.1186/s12966-020-00975-y)
Supplement: Supplementary file 2 — Additional file 2. Participant flowchart for the Measuring EAting in Everyday Life Study. [file 12966_2020_975_MOESM2_ESM.docx]

**Additional File 2.** Participant flowchart for the Measuring EAting in Everyday Life Study

*Includes participants who had at least one entry of dietary data in their allocated recording days
