# Supplementary material for: Eating occasion situational factors and sugar-sweetened beverage consumption in young adults
Source: Int J Behav Nutr Phys Act. 2020 Jun 3;17:71. doi: 10.1186/s12966-020-00975-y (PMC7271392; doi:10.1186/s12966-020-00975-y)
Supplement: Supplementary file 3 — Additional file 3. A checklist of items included in the present manuscript as recommended by the extended STROBE-nut statement for nutritional observational studies [1, 2]. [file 12966_2020_975_MOESM3_ESM.doc]

**Additional File 3:** A checklist of items included in the present manuscript as recommended by the extended STROBE-nut Statement for nutritional observational studies [1, 2].

|  | Item No | Recommendation | | Present:  Yes/No/NA | Page No (Line No) |
| --- | --- | --- | --- | --- | --- |
| **Title and abstract** | Nut 1 | (a) State the dietary/nutritional assessment methods in the title, abstract or key words | | Yes | 1 (28-30; 54) |
|  |  | (*b*) Provide in the abstract an informative and balanced summary of what was done and what was found | | Yes | 1 (27-47) |
| **Introduction** |  |  | |  |  |
| Background/  rationale | 2 | Explain the scientific background and rationale for the investigation being reported | | Yes | 3-4 |
| Objectives | 3 | State specific objectives, including any prespecified hypotheses | | Yes | 4 (98-100) |
| **Methods** |  |  | |  |  |
| Study design | 4 | Present key elements of study design early in the paper | | Yes | 5 (104-114) |
| Setting | 5 | Describe the setting, locations, and relevant dates, including periods of recruitment, exposure, follow-up, and data collection  Nut-5 Describe any characteristics of the study settings that might affect the dietary intake or nutritional status of the participants, if applicable. | | Yes | 5-6 (104-144) |
| Participants | 6 | (*a*) *Cohort study*—Give the eligibility criteria, and the sources and methods of selection of participants. Describe methods of follow-up  *Case-control study*—Give the eligibility criteria, and the sources and methods of case ascertainment and control selection. Give the rationale for the choice of cases and controls  *Cross-sectional study*—Give the eligibility criteria, and the sources and methods of selection of participants | | Yes | 4 (105-107) |
|  |  | (*b*) *Cohort study*—For matched studies, give matching criteria and number of exposed and unexposed  *Case-control study*—For matched studies, give matching criteria and the number of controls per case | | NA |  |
|  | Nut 6 | Report particular dietary, physiological or nutritional characteristics that were considered when selecting the target population. | | Yes | 4 (105) |
| Variables | 7 | Clearly define all outcomes, exposures, predictors, potential confounders, and effect modifiers. Give diagnostic criteria, if applicable | | Yes | 6-7 |
|  | Nut 7.1 | Clearly define all foods, food groups, nutrients or other food components. | | Yes | 6-7 |
|  | Nut 7.2 | When using dietary patterns or indices, describe the methods used to obtain them and their nutritional properties. | | NA |  |
| Data sources/ measurement | 8* | For each variable of interest, give sources of data and details of methods of assessment (measurement). Describe comparability of assessment methods if there is more than one group | | Yes | 5-8 |
|  | Nut 8.1 | Describe the Dietary Assessment Method(s), E.g., Portion Size Estimation, Number of Days and Items Recorded, How It Was Developed and Administered, and How Quality Was Assured. Report If and How Supplement Intake Was Assessed | | Yes | 6-7 (116-144) |
|  | Nut 8.2 | Describe and Justify Food Composition Data Used. Explain the Procedure to Match Food Composition with Consumption Data. Describe the Use of Conversion Factors, If Applicable | | Yes | 6 (138-144) |
|  | Nut 8.3 | Describe the Nutrient Requirements, Recommendations, or Dietary Guidelines and the Evaluation Approach Used to Compare Intake with the Dietary Reference Values, If Applicable | | NA |  |
|  | Nut 8.4 | When Using Nutritional Biomarkers, Additionally Use the STROBE Extension for Molecular Epidemiology (STROBE-ME). Report the Type of Biomarkers Used and Usefulness as Dietary Exposure Markers | | NA |  |
|  | Nut 8.5 | Describe the Assessment of Nondietary Data (E.g., Nutritional Status and Influencing Factors) and Timing of the Assessment of These Variables in Relation to Dietary Assessment | | Yes | 7 (159-170) |
|  | Nut 8.6 | Report on the Validity of the Dietary or Nutritional Assessment Methods and Any Internal or External Validation Used in the Study, If Applicable | | Yes | 6 (134-137) |
| Bias | 9 | Describe any efforts to address potential sources of bias | | Yes | 9 (201-213)  15 (349-354) |
|  | Nut 9 | Report How Bias in Dietary or Nutritional Assessment, E.g., Misreporting, Changes in Habits as a Result of Being Measured, and Data Imputation from Other Sources, Was Addressed | | Yes | 6 (129-137) |
| Study size | 10 | Explain how the study size was arrived at | | Yes | 8 (184-189) |
| Quantitative variables | 11 | Explain how quantitative variables were handled in the analyses. If applicable, describe which groupings were chosen and why | | Yes | 7-8 (156-158; 163-183)  9 (202-206) |
|  | Nut 11 | Explain the Categorization of Dietary/Nutritional Data (E.g., Use of N-tiles and Handling of Nonconsumers) and the Choice of Reference Category, If Applicable | | NA |  |
| Statistical methods | 12 | (*a*) Describe all statistical methods, including those used to control for confounding | | Yes | 8-9 (191-216) |
|  |  | (*b*) Describe any methods used to examine subgroups and interactions | | Yes | 8-9 (193-200) |
|  |  | (*c*) Explain how missing data were addressed | | Yes | 8 (186-189)  15 (349-353) |
|  |  | (*d*) *Cohort study*—If applicable, explain how loss to follow-up was addressed  *Case-control study*—If applicable, explain how matching of cases and controls was addressed  *Cross-sectional study*—If applicable, describe analytical methods taking account of sampling strategy | | NA |  |
|  |  | (*e*) Describe any sensitivity analyses | | NA | - |
|  | Nut 12.1 | Describe Any Statistical Method Used to Combine Dietary or Nutritional Data, If Applicable | | NA |  |
|  | Nut 12.2 | Describe and Justify the Method for Energy Adjustments, Intake Modeling and Use of Weighting Factors, If Applicable | | NA |  |
|  | Nut 12.3 | Report Any Adjustments for Measurement Error, I.e., from a Validity or Calibration Study | | NA |  |
| **Results** |  | |  |  |  |
| Participants | 13* | | (a) Report numbers of individuals at each stage of study—eg numbers potentially eligible, examined for eligibility, confirmed eligible, included in the study, completing follow-up, and analysed | Yes | 8 (184-189)  Additional File 2 |
|  |  |  | (b) Give reasons for non-participation at each stage | Yes | Additional File 2 |
|  |  |  | (c) Consider use of a flow diagram | Yes | Additional File 2 |
|  | Nut 13 | | Report the Number of Individuals Excluded Based on Missing, Incomplete, or Implausible Dietary/Nutritional Data | Yes | 8 (184-189)  Additional File 2 |
| Descriptive data | 14* | | (a) Give characteristics of study participants (eg demographic, clinical, social) and information on exposures and potential confounders | Yes | 9-10 (217-223); Table 1 (27) |
|  |  |  | (b) Indicate number of participants with missing data for each variable of interest | Yes | 27, 29-31 (Table footnotes) |
|  |  |  | (c) *Cohort study*—Summarise follow-up time (eg, average and total amount) | NA |  |
|  | Nut 14 | | Give the Distribution of Participant Characteristics across the Exposure Variables If Applicable. Specify If Food Consumption of Total Population or Consumers Only Were Used to Obtain Results | Yes | 27 (Table 1) |
| Outcome data | 15* | | *Cohort study*—Report numbers of outcome events or summary measures over time | NA |  |
|  |  |  | *Case-control study—*Report numbers in each exposure category, or summary measures of exposure | NA |  |
|  |  |  | *Cross-sectional study—*Report numbers of outcome events or summary measures | Yes | 27-28 (Tables 1 & 2) |
| Main results | 16 | | (*a*) Give unadjusted estimates and, if applicable, confounder-adjusted estimates and their precision (eg, 95% confidence interval). Make clear which confounders were adjusted for and why they were included | Yes | 29-31 (Tables 3-5) |
|  |  |  | (*b*) Report category boundaries when continuous variables were categorized | Yes | 8 (175-176; 182-183) |
|  |  |  | (*c*) If relevant, consider translating estimates of relative risk into absolute risk for a meaningful time period | NA |  |
|  | Nut 16 | | Specify If Nutrient Intakes Are Reported with or without Inclusion of Dietary Supplement Intake, If Applicable | NA |  |
| Other analyses | 17 | | Report other analyses done—eg analyses of subgroups and interactions, and sensitivity analyses | NA |  |
| Discussion |  | |  |  |  |
| Key results | 18 | | Summarise key results with reference to study objectives | Yes | 11-14 |
| Limitations | 19 | | Discuss limitations of the study, taking into account sources of potential bias or imprecision. Discuss both direction and magnitude of any potential bias | Yes | 15 (342-354) |
|  | Nut 19 | | Describe the Main Limitations of the Data Sources and Assessment Methods Used and Implications for the Interpretation of the Findings | Yes | 15 (342-354) |
| Interpretation | 20 | | Give a cautious overall interpretation of results considering objectives, limitations, multiplicity of analyses, results from similar studies, and other relevant evidence | Yes | 15 (355-363) |
|  | Nut 20 | | Report the Nutritional Relevance of the Findings, Given the Complexity of Diet or Nutrition as an Exposure | Yes | 15 (358-360) |
| Generalisability | 21 | | Discuss the generalisability (external validity) of the study results | Yes | 15 (345-351) |
| **Other information** |  | |  |  |  |
| Funding | 22  Nut 22.1  Nut 22.2 | | Give the source of funding and the role of the funders for the present study and, if applicable, for the original study on which the present article is based  Describe the Procedure for Consent and Study Approval from Ethics Committee(s)  Provide Data Collection Tools and Data as Online Material or Explain How They Can Be Accessed | Yes  Yes  Yes | 17 (386-392)  16 (374-377)  16 (380-382) |

1. von Elm E, Altman DG, Egger M, Pocock SJ, Gotzsche PC, Vandenbroucke JP: **The Strengthening the Reporting of Observational Studies in Epidemiology (STROBE) Statement: guidelines for reporting observational studies**.

2. Lachat C, Hawwash D, Ocké MC, Berg C, Forsum E, Hörnell A, et al.: Strengthening the Reporting of Observational Studies in Epidemiology—

Nutritional Epidemiology (STROBE-nut): An Extension of the STROBE Statement. PLoS Med 2016, **13**(6).

*Give information separately for cases and controls in case-control studies and, if applicable, for exposed and unexposed groups in cohort and cross-sectional studies.
